# Associations of Environmental Features With Outdoor Physical Activity on Weekdays and Weekend Days: A Cross-Sectional Study Among Older People

**DOI:** 10.3389/fpubh.2020.578275

**Published:** 2020-10-30

**Authors:** Kirsi E. Keskinen, Ying Gao, Merja Rantakokko, Taina Rantanen, Erja Portegijs

**Affiliations:** ^1^Faculty of Sport and Health Sciences and Gerontology Research Center, University of Jyväskylä, Jyväskylä, Finland; ^2^School of Resource Wisdom, University of Jyväskylä, Jyväskylä, Finland; ^3^Department of Sports Science, College of Education, Zhejiang University, Hangzhou, China; ^4^School of Health and Social Studies, JAMK University of Applied Sciences, Jyväskylä, Finland

**Keywords:** aging, walking, mobility, GIS, day-to-day variability

## Abstract

**Background:** Physical activity (PA) of higher intensity and longer duration mainly accumulates from older adults' out-of-home activities. Outdoor PA is influenced by environmental features; however, the day-to-day variability of PA and its associations with environmental features have not been widely studied. This study focused on the associations of environmental features with accelerometer-measured PA in older people on weekdays and weekend days.

**Methods:** The study population comprised 167 community-dwelling older people aged 75–90 years. Accelerometers were worn on 7 consecutive days and a structured interview on physical functioning, health, and socioeconomic factors was administered. A geographic information system (GIS) was used to assess environmental features within a distance of 500 (number of land types, road network slope, intersection, and residential densities) or 1,000 m (habitat diversity within natural and green areas) from participants' homes. Accelerometer-based PA [number of PA bouts >10 min and minutes of moderate to vigorous physical activity (MVPA)] was analyzed for weekdays and weekend days separately. Associations between environmental features and PA were analyzed using linear regression models.

**Results:** Participants accumulated on average 0.60 PA bouts and 34.2 MVPA minutes on weekdays and 0.50 PA bouts and 31.5 MVPA minutes on weekend days. Especially participants with low overall PA were less active at weekends. Habitat diversity in natural and green areas, intersection density, and residential density were positively associated with numbers of PA bouts and MVPA minutes on weekdays. Moreover, more diversity in natural and green areas was associated with more MVPA minutes on weekend days. A higher road network slope was negatively associated with the number of PA bouts throughout the week and with MVPA minutes on weekend days.

**Conclusions:** Environmental features close to home, especially PA-supportive infrastructural features and services, were more strongly associated with weekday than weekend PA. This suggests that older people's out-of-home activities, typically conducted on weekdays, are related to service use. However, greater diversity of natural areas close to home seemed to motivate older adults to engage in higher MVPA throughout the week.

## Introduction

Older adults are recommended to engage in moderate to vigorous intensity (MVPA) for at least 150 min a week (1). For older people, a large proportion of their physical activity (PA) accumulates during daily activities, such as walking for transport, and is not necessarily exercise-related (2). Then again, transportation walking may be a form of daily exercise for some older adults or be combined with walking for leisure, which makes categorizing of older adults' PA challenging. Either way, out-of-home activities are associated with higher PA, especially when moving through greater life-space areas (3). A previous study investigating the day-to-day variability of PA found significant differences between weekdays and weekend days in time spent on PA and concluded that these differences in habitual PA were probably explained by daily routines and practices (4). It is not clear whether environmental features in the home neighborhood relate to PA accumulation similarly on weekdays and weekend days among older adults.

For Finnish older adults, shopping, walking for exercise, social visits, and running errands are among the commonest reasons for going outside the home (5). The extent to which neighborhoods and cities offer such destinations and are conducive to PA varies greatly (6) and thus is not the same for all older adults. Based on a large international study, the difference between the least and the most activity-supporting urban environment could mean a difference of more than 60 min in weekly MVPA among adults (6). Recent meta-analyses have showed positive associations between multiple environmental features and PA (7), walking for transport (8), and leisure-time PA (9). It has also been observed that physical functioning (10) and socioeconomic (11) status may be intertwined with associations between objectively defined features of the environment and PA. However, given the variability in daily routines and in the availability of services by the day of the week, environmental features associated with PA may differ across days of the week. To learn more about age-friendly environments calls for information on individuals' health behavior in space and time (12).

Features such as street connectivity, residential density, and mixed land use, whether as separate environmental features or in combination to form a walkability index, are indicative of service availability in the environment, and have all been positively associated with time spent on MVPA (2, 10, 11, 13, 14), although not consistently (15–18). MVPA has shown positive associations with closeness of parks (19) and density of recreation facilities (8), yet the associations have also appeared as non-significant (10, 13). Furthermore, research indicates that for older adults, walking to a daily destination typically takes at least 10 min (20). Thus, to include habitual outdoor activities, it may be necessary to capture continuous bouts of PA lasting at least 10 min. Bouts of at least 10 min have for a long time considered beneficial for health; however, current PA guidelines acknowledge the benefit of any activity and any breaks in sedentary time, regardless of their duration (1).

With declining function, older adults become more vulnerable to environmental barriers (21). Consequently, older adults may modify their behavior, e.g., by resting in the middle of a walk with steep slopes in the immediate home environment (22)—in other words, by shortening their activity bouts. Negative associations between hilly terrain and walking (23), total PA (24), and recreational PA (25) have been reported, although non-significant relationships with leisure-time PA have also been found (26). Nevertheless, to the best of our knowledge, the associations between hilly terrain and PA bouts and MVPA in older adults have not been studied.

The purpose of this study was to gain more understanding on variability in PA levels between weekdays and weekend days and, especially, on which environmental features may support habitual PA of older adults and when. For that, we examined the PA levels on weekdays and weekend days and explored the associations of environmental features with the number of PA bouts and MVPA minutes on weekdays and weekend days in older adults. In addition, by applying a method similar to that used by Sallis et al. (6), we aimed to estimate whether differences in the extent of environmental features supporting PA in a neighborhood would show practical relevance for older adults' PA levels estimated as the number of PA bouts and MVPA minutes. We included environmental features conducive to PA that are related to performing daily errands (intersection density and residential density) and engaging in recreational activities (number of land types, habitat diversity in green areas), and features hindering PA (hilly terrain). Land type, habitat diversity, and slope are also among the natural elements in a neighborhood. Intersection and residential density, in turn, are features of walkability, and thus indicate the amount of infrastructure supporting outdoor mobility (27).

## Materials and Methods

### Study Design

This study is part of the project “Geographic characteristics, outdoor mobility and physical activity in old age” (GEOage) (28). In the project, data on participants' PA were combined with data on the environmental characteristics of their home surroundings. The participant data had earlier been collected in the project “Life-space mobility in old age” (LISPE), which has been described in detail elsewhere (29). For the LISPE study, a random sample of 2,550 community-dwelling older people aged 75–90 living in the neighboring municipalities of Jyväskylä and Muurame in Central Finland, which thereby formed the study area, was drawn from the population register in the winter of 2011/2012. Of these, 848 fulfilled the eligibility criteria (living independently in the recruitment area, being able to communicate, and willing to participate in the study) and were interviewed in their homes, using a structured questionnaire, in spring 2012. Participants signed a written informed consent before the home interview. The ethical committee of the University of Jyväskylä, Finland approved the LISPE and GEOage projects.

Research staff took an accelerometer to the home interview whenever one was available. After completion of the home interview, the interviewer asked verbally whether the participant was willing to participate in the accelerometer sub-study. Based on participant willingness and accelerometer availability, a sub-sample of 190 participants was assigned to wear a tri-axial accelerometer (Hookie AM20 Activity Meter; Hookie Technologies Ltd, Espoo, Finland) for 7 consecutive days following the home interview. The participants were instructed to wear the accelerometer (size 6.6 × 2.7 × 1.3 cm, mass 15 g), which was attached to an elastic belt, on their right hip during waking hours. They were told to take off the accelerometer only when engaging in activities, in which the accelerometer would get into contact with water. After the measurement period, participants returned the accelerometer in a prepaid envelope by mail, or in some cases, the accelerometer was picked up from their home. Data from 16 participants were excluded due to technical problems ending the accelerometer recording abruptly (*n* = 3), the accelerometer being lost in the mail (*n* = 1), accelerometer wear time not meeting the criterion of at least 10 h per day (*n* = 11), and intermissions of more than 1 day between consecutive measurement days (*n* = 1). Of the remaining participants, only those with valid measurement data for at least 2 weekdays (Monday–Friday) and at least one weekend day (Saturday–Sunday) were included in the study (excluded *n* = 7). This resulted in a final sample of 167. On average, those in the accelerometer sub-sample less frequently reported difficulties in walking 500 m (15% of sub-sample participants) than all the LISPE participants combined [26%, $\chi_{\left(1\right)}^{2}$ = 8.09, *p* < 0.05]. Otherwise no differences were observed between the two samples in mean age, proportion of women, years of education, or number of chronic conditions (for all *p* ≥ 0.269). Those who agreed to participate in the LISPE study were younger, more often lived alone, perceived their health as at least moderate, perceived fewer difficulties in outdoor mobility, and more frequently moved outside daily than those who declined to participate (29).

In the GEOage project, participants' homes, addresses for which were retrieved from the population register, were located on a map (30) using a geographic information system (GIS) (ArcMap version 10.3; Esri, Redlands, CA, USA). Openly available geospatial datasets were imported to the GIS to characterize the environment in the study area and within 500 or 1,000 m from participants' homes. In general, the study area is characterized by lakes, forest, low hills, and relatively continuous areas of built environment surrounded by sparsely populated areas. In the year 2012, the population of the study area was 143,000 inhabitants (31), with the majority concentrated in and around the center areas.

### Study Measures

#### Physical Activity

Participants' PA was objectively assessed by an accelerometer (Hookie AM20 Activity Meter; Hookie Technologies Ltd, Espoo, Finland). The accelerometer records accelerations along three axes, x, y, and z, i.e., vertical, horizontal, and perpendicular, respectively, and has a dynamic range of ±16 g, 13 bits at 100 Hz. With accelerometer raw data available for our use, the resultant acceleration of each recorded sample was calculated and used in all further analyses. Mean amplitude deviation (MAD) (32) was calculated in non-overlapping 5-s epochs and subsequently averaged in 1-min epochs, using a custom-written Matlab script (R2015b, Mathworks, Inc., Natick, MA, USA). The pre-processed 1-min data were divided into 24-h segments from midnight to midnight, and further processing was done in those 24-h segments. Non-wear time was defined as any continuous epochs lasting at least 1 h with all the 1-m MADs below 0.024 g. This non-wear algorithm produced results congruent with self-reported accelerometer non-wear time.

PA was assessed from the 1-m MAD epoch values for each of the 24-h segments. The 1-m values were classified into sedentary (<0.0167 g), light PA (0.0167 to <0.091 g), moderate PA (0.091 to <0.414 g), or vigorous PA (≥0.414 g) after excluding all non-wear minutes. The intensity cut-offs were based on the optimal classification for light PA (0.0167 g) (32), and at MADs corresponding to 3 metabolic equivalents (MET, 0.091 g), and 6 METs (0.414 g) for moderate PA and vigorous PA, respectively (33). MVPA minutes was the sum of the minutes spent in moderate PA and vigorous PA. The accumulation of PA bouts was assessed based on the 1-m epochs of light PA and MVPA (34) and all active bouts lasting >10 min were counted (35). From the number of PA bouts lasting >10 min and total MVPA minutes in each 24-h segment, overall values were calculated as the mean of all the 24-h segments, weekday values as the mean of the 24-h segments from Monday to Friday, and weekend day values as the mean of the 24-h segments from Saturday to Sunday for both the number of PA bouts and MVPA minutes. Similarly, accelerometer wear time overall, on weekdays, and on weekend days was calculated as the mean value of the respective 24-h segments.

#### Environmental Features

Number of land types [*n*] was recorded as counts of different land types within a 500-m radius of the participant's home (36). The original 48 land type classes in the Corine Land Cover dataset (37) were reclassified into three built and 10 natural environment land types (38). Thus, the value of the variable reflects the variation present especially in the natural environment surrounding the participants' homes.

Habitat diversity in natural and green areas [index, range 0…10] was defined as the highest normalized value of the Shannon's Diversity Index (SHDI) (36) among natural and green areas, which were of a minimum size of 10 hectares and located at least partly within 1,000 m from participants' homes. We chose a 1,000-m radius for this variable, as previous studies have shown that greenness (39, 40) and attractive destinations (41) at longer distances from the home may be positively associated with parameters of health and PA. To capture the diversity of the participants' natural environments, SHDI values were calculated only considering the nine natural environment land types, excluding water, included in the reclassified Corine Land Cover data (37). To enable meaningful interpretation of the results, the final SHDI value was calculated by multiplying the original index values (range 0–1) by 10.

Road network slope was defined as the average slope [% rise] (where 1% point equals a gradient of 0.45 degrees) in the 500-m road network (42) of each participant. We used the Digital Elevation Model available in the 2 m × 2 m raster dataset (43) to calculate slope values for every 2-m section of the roads in the study area. A participant's road network slope was the mean of the road section slope values in the 500-m road network.

Intersection density [10 intersections/km^2^] was calculated as the number of intersections within a 500-m radius of the participant's home divided by the surface area of this zone. To enable meaningful interpretation of the results, this number was subsequently divided by 10. An intersection was defined as the junction of a minimum of three roads, and intersections within a distance of 10 m from one another were merged. In calculating this variable, we used the road data in the Topographic Database 2013 (42).

Residential density [1,000 residents/km^2^] was defined as average residential density within a 500-m radius of the participant's home. For meaningful interpretation, this number was divided by 1,000. To calculate mean values, we first transformed the original 1 km × 1 km grid drawn from the Population grid data 2012 (44) into finer cells of 100 m × 100 m.

#### Covariates

To account for socio-demographic differences (7, 45), age, sex, and years of education were used as covariates in the analyses. Age and sex were retrieved from the national population register. Completed years of education was ascertained during the home interview. No imputation was made for the three missing cases. The moderating effects of the participants' physical functioning on the association between the built environment and physical activity (46–48) were evaluated with the variables walking difficulties and number of chronic conditions. Difficulties in walking 500 m were ascertained during the interview, and those reporting at least some difficulties were assigned to the category of perceived walking difficulties (vs. no walking difficulties). Self-reported number of chronic conditions was summed based on a list of 22 physician-diagnosed chronic conditions and an additional open-ended question (29).

### Analysis

Participants and their environments were characterized by means, standard deviations and ranges, by medians and interquartile ranges (IQR), or by percentages depending on variable distribution. Distributions of PA variable values between weekdays and weekend days were compared using the Wilcoxon signed-rank test and Pearson chi-square test. Bland-Altman plots with 95% confidence intervals for differences in mean values between individuals' weekday and weekend day PA, including regression lines (49), were created for PA bouts and MVPA time. Bland-Altman plots are especially suited for visually detecting differences in corresponding values from repeated measurements or from two measurement methods along the measurement scale (50).

The PA variables showed right-skewed distributions and, except for mean MVPA on weekdays, included a substantial proportion of zero values. Hence, for the further analysis, the PA variable values were transformed using a natural logarithm after adding the value of one to remove zeros. Associations of each environmental variable with each of the log-transformed PA variables were studied using linear regression. Associations were adjusted first for age, sex, and accelerometer wear time (Model 1), and then—one at a time due to the relatively small sample size—for perceived difficulties in walking 500 m (Model 2), years of education (Model 3), and chronic conditions (Model 4). To interpret the results, the values of coefficients and confidence intervals from the log-transformed linear regression analyses were exponentiated (marked as exp^β^). In the result tables, the reported exp^β^ value equals the proportional change in the outcome variable value obtained from a one-unit increase in the predictor value. Additional sensitivity analyses were performed by rerunning the analyses with a sample restricted to participants with valid data for 3 or more weekdays and both weekend days (*n* = 139).

To estimate the magnitude of environmental effects on PA, we performed calculations using the results of the Model 2 linear regression analyses, the high-low difference in environmental variable values within the study area, and the median values of the number of PA bouts and MVPA minutes overall. Coefficients and confidence intervals of environmental variables showing statistically significantly associations with PA were multiplied by the value of the high-low difference, which was defined as the difference between the means of the highest and lowest 10% of the environmental variable values. This product was then exponentiated to show the proportional effect on PA of the high-low difference for the environmental variable in question. Furthermore, to express the absolute effects of the high-low difference for the environmental variables in the number of PA bouts and MVPA minutes, the proportional effect values were multiplied by the respective PA median values.

Data analyses were performed with IBM SPSS Statistics for Windows (version 24.0; IBM Corp., Armonk, NY, USA) and R software version 3.5.2 (51). Statistical significance was set at *p* < 0.05 in all tests.

## Results

Participant and neighborhood characteristics are presented in Table 1. Participant mean age was 80.3 years, 65% were women, and 15% perceived walking difficulties (Table 1).

**Table 1:** Characteristics of participants and their neighborhood environments (*n* = 167).

	**Mean**	**(Standard**	**Range**
		**deviation)**	**Min–Max**
**Participant characteristics**			
Age [years]	80.3	(4.2)	74.3–89.3
Education [years]	9.9	(4.0)	2–25
Chronic conditions [n]	4.3	(2.3)	0–11
Women [%]	65		
Walking difficulties [%]	15		
**Neighborhood characteristics**			
Land types [*n*]	6.1	(1.2)	4–9
Habitat diversity [10*SHDI]	4.3	(1.2)	1.3–7.1
Slope [% rise]	2.1	(0.6)	1.1–3.9
Intersection density [10 crossings/km^2^]	5.9	(2.4)	0.5–10.9
Residential density [1,000 residents/km^2^]	1.8	(1.5)	0.002–5.0

### Variability in Physical Activity Levels Between Weekdays and Weekend Days

The median number of accumulated PA bouts on weekdays and weekend days were similar (*p* = 0.646), with values below 1 indicating that the majority of the participants did not engage in a 10-min bout of continuous movement every day (Table 2). The median time spent in MVPA was about half an hour and did not differ between weekdays and weekend days (*p* = 0.125). On average, participants wore the accelerometer for a longer time on weekdays than weekend days (*p* < 0.001). The number of participants accumulating zero PA bouts was higher on weekend days (72 participants) than weekdays (43 participants) [$\chi_{\left(1\right)}^{2}$ = 48.366, *p* < 0.001].

**Table 2:** Accelerometer-derived PA and wear time overall (all days) and on weekdays and weekend days (*n* = 167).

	**Overall**		**Weekdays**		**Weekend days**		**Wilcoxon signed-rank test^**a**^**
	**Median**	**(IQR)**	**Median**	**(IQR)**	**Median**	**(IQR)**	***p*-value**
PA bouts [n/day]	0.57	(0.14, 1.33)	0.60	(0.00, 1.33)	0.50	(0.00, 1.50)	0.646
MVPA [min/day]	31.3	(16.7, 54.4)	34.2	(17.8, 56.4)	31.5	(10.5, 50.0)	0.125
Accelerometer wear time [h/day]	13.5	(12.7, 14.3)	13.7	(12.7, 14.5)	13.2	(12.3, 14.2)	**<0.001**

a *Comparison between weekday and weekend day values. Values in bold; p < 0.05. IQR, Interquartile range (25%, 75%)*.

**Figure 1:**
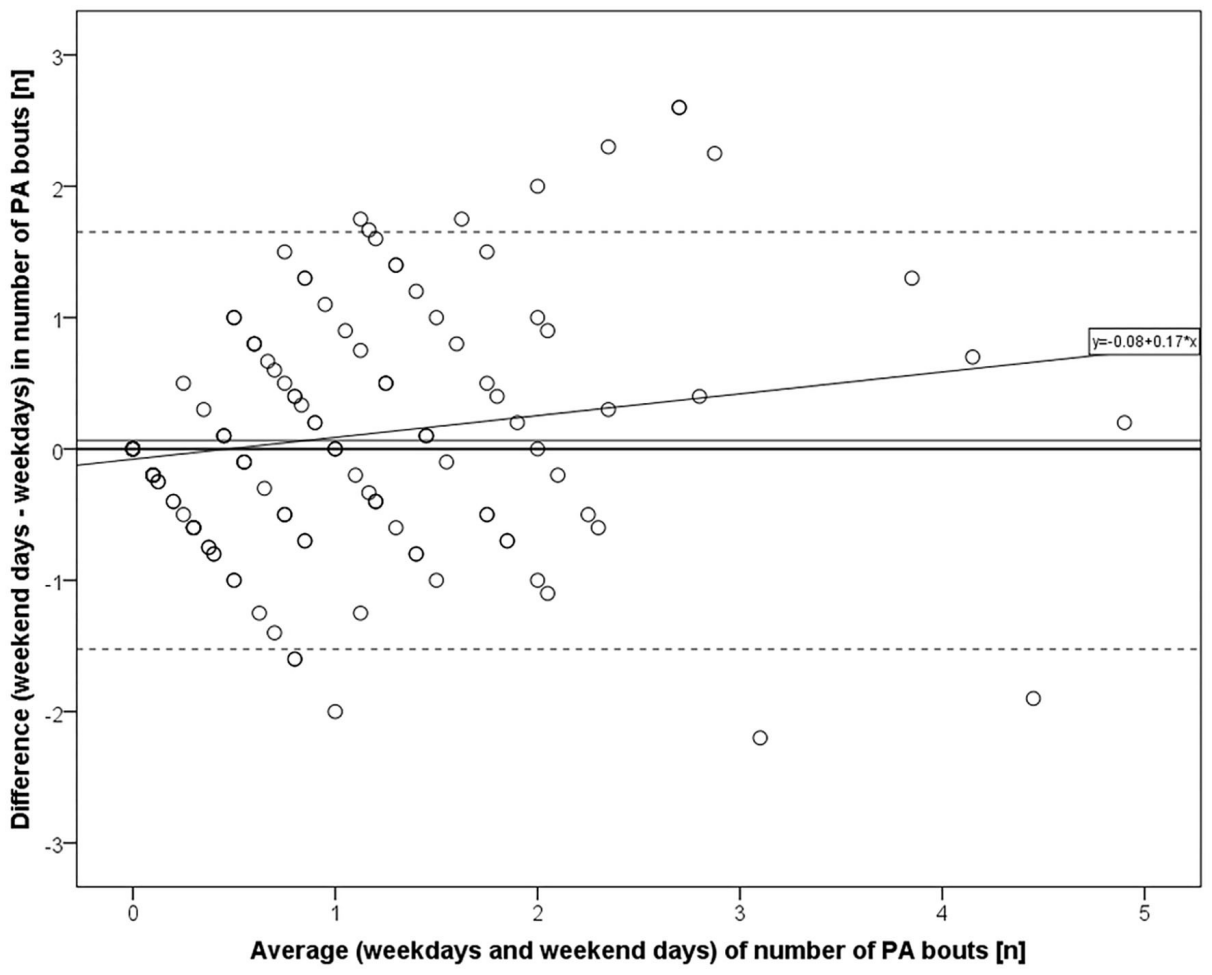
Bland-Altman plot for number of PA bouts on weekend days vs. weekdays (*n* = 167). Mean difference with 95% confidence intervals and regression line (*R*^2^ = 0.036, *p* = 0.014 for β).

**Figure 2:**
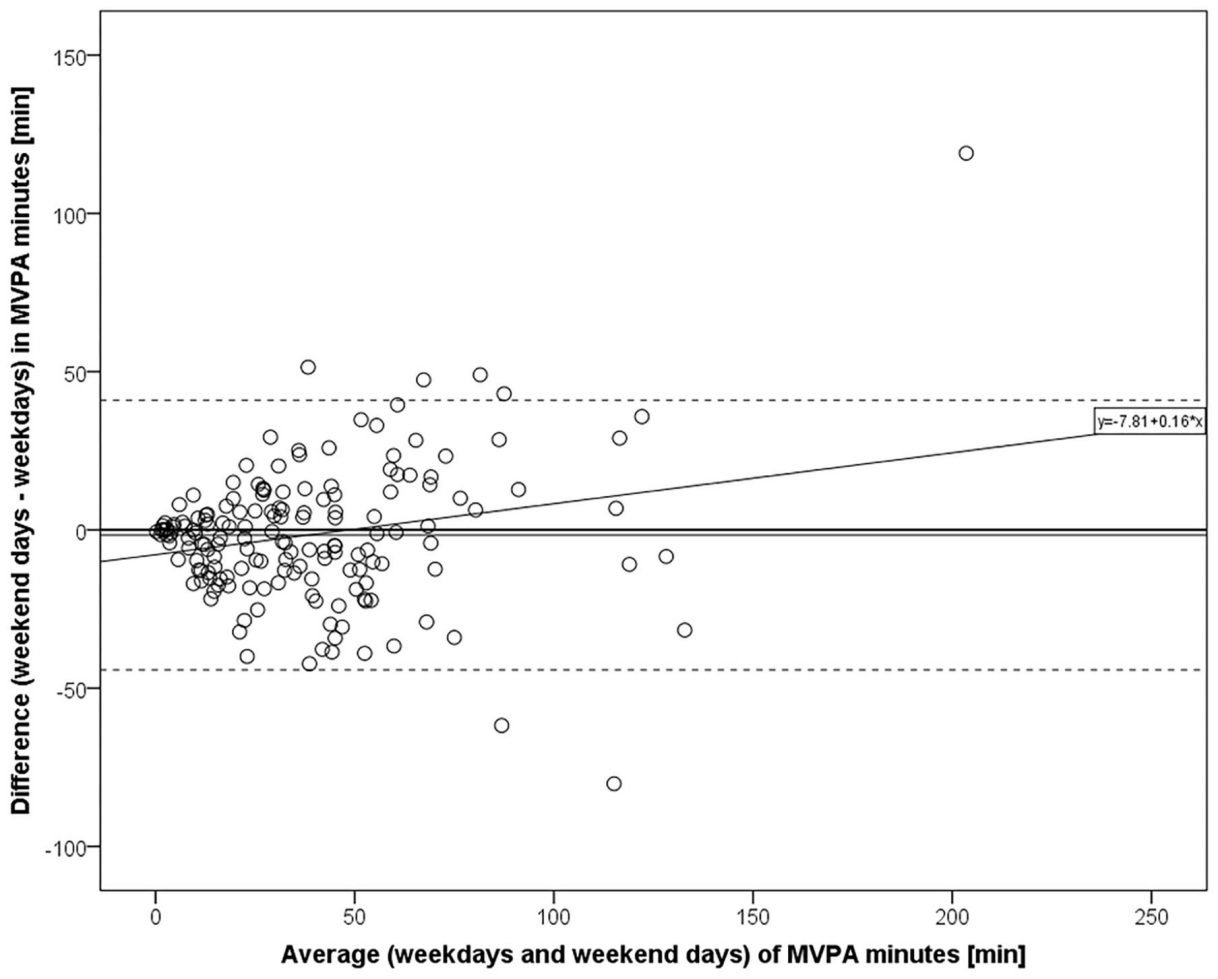
Bland-Altman plot for MVPA minutes on weekend days vs. weekdays (*n* = 167). Mean difference with 95% confidence intervals and regression line (*R*^2^ = 0.052, *p* = 0.003 for β).

The Bland-Altman plot data on individuals showed that the participants accumulated on average 0.06 more PA bouts [*t*_(166)_ = 1.015, *p* = 0.311] and 1.64 fewer MVPA minutes [*t*_(166)_ = −0.972, *p* = 0.332] on weekend days compared to weekdays; however, these mean differences did not statistically significantly differ from zero. However, participants with lower PA in general often exhibited negative differences, indicating greater activity on weekdays compared to weekend days (Figures 1, 2).

### Environmental Features Associated With Physical Activity on Weekdays and Weekend Days

The linear regression analyses adjusted for age, sex, average accelerometer wear time, and walking difficulties yielded different environment-PA associations between weekdays and weekend days (Table 3). On weekdays, habitat diversity in natural and green areas, intersection density, and residential density were positively associated with both the number of PA bouts and MVPA minutes. On weekend days, only habitat diversity was positively associated with MVPA and had a slightly lower coefficient value than on weekdays. Road network slope showed a negative association with PA bouts on weekdays and weekend days and with MVPA on weekend days only. Number of land types was not associated with PA in any of the analyzed models. Additional File 1 also shows the results of the models in which associations were adjusted for age, sex, and accelerometer wear time (Model 1), and also for years of education (Model 3), and chronic conditions (Model 4). No substantial differences in results were observed between the models, except for slope, which was associated with MVPA only in the model adjusted for walking difficulties (Model 2). The results of rerunning the models in the sensitivity analyses by including participants with valid accelerometer data for at least 5 days largely resembled those of the full sample, with some associations in the full sample with *p* < 0.100 reaching statistical significance and some associations turning statistically non-significant (*p*-value between 0.050 and 0.100) (see Additional File 2).

**Table 3:** Associations of environmental features with PA bouts and MVPA on weekdays and weekend days (*n* = 167).

	**Number of PA bouts**		**MVPA minutes**	
	**Weekdays**	**Weekends**	**Weekdays**	**Weekends**
	**exp^**β**^ (95% CI)**	**exp^**β**^ (95% CI)**	**exp^**β**^ (95% CI)**	**exp^**β**^ (95% CI)**
Land types [*n*]	1.00 (0.94–1.05)	1.04 (0.98–1.10)	0.99 (0.89–1.09)	1.05 (0.94–1.17)
Habitat diversity [10*SHDI]	**1.07 (1.01–1.12)**	1.02 (0.97–1.08)	**1.16 (1.06–1.28)**	**1.11 (1.00–1.23)**
Slope [% rise]	**0.86 (0.77–0.96)**	**0.85 (0.75–0.97)**	0.82 (0.66–1.01)	**0.78 (0.62–0.98)**
Intersection density [10 crossings/km^2^]	**1.03 (1.01–1.06)**	1.01 (0.98–1.05)	**1.07 (1.01–1.12)**	1.03 (0.97–1.09)
Residential density [1,000 residents/km^2^]	**1.09 (1.04–1.13)**	1.03 (0.98–1.08)	**1.15 (1.06–1.25)**	1.08 (0.99–1.18)

*Results of Model 2; adjusted for age, sex, accelerometer wear time, and difficulties in walking 500 m. Values in bold; p < 0.05. Antilogarithm values of unstandardized regression coefficients (exp^β^) and their 95% confidence intervals (CI) from univariate linear regression models show a proportional effect of a one-unit increase in the predictor value on the outcome variable value (e.g., a one-unit increase in residential density, equaling an increase of 1,000 residents in a 1-km^2^ area, shows a 9% increase in the number of PA bouts and 15% increase in MVPA minutes on weekdays)*.

### Effects of High-Low Differences in Environmental Features on Physical Activity

The proportional and absolute effects on PA of the high-low differences in environmental features in the study area are shown in Table 4. The effects were estimated in those environmental features, which were statistically significantly associated with number of PA bouts and MVPA minutes overall on all days. The highest effects were detected in residential density, in which the high-low difference of 4.55 thousand people per km^2^ resulted in an increase of 36% in the number of PA bouts and 74% in MVPA minutes overall (proportional effects), which corresponded to 0.21 more PA bouts and 23.1 more MVPA minutes daily (absolute effects). Altogether, the proportional effects of the environmental variables positively associated with PA varied between 21 and 36% for PA bouts and between 57 and 74% for MVPA minutes. Depending on the high-low difference in the environmental feature in question, these resulted in absolute effects between 0.12 and 0.21 more PA bouts and between 18.0 and 23.1 more MVPA minutes daily. Slope was the only environmental feature showing a negative effect, with the high-low difference resulting in a 26% decrease in PA bouts and 33% in MVPA, equivalent to 0.15 fewer PA bouts and 10.3 fewer MVPA minutes daily.

**Table 4:** Proportional and absolute effects of high-low differences in environmental variables on PA (*n* = 167).

**Environmental variable**	**High-low difference [in environmental variable units]**	**exp^**β**^ (95% CI)^**a**^**	**Proportional effect (95% CI)**	**Absolute effect [in units of PA variable]**
**Number of PA bouts overall [n/day]**				
Land types [*n*]	4.12	1.01 (0.96–1.06)		
Habitat diversity [10*SHDI]	3.84	**1.05 (1.00–1.10)**	1.21 (1.01–1.45)	0.12
Slope [% rise]	1.92	**0.86 (0.77–0.95)**	0.74 (0.61–0.91)	−0.15
Intersection density [10 crossings/km^2^]	8.37	**1.03 (1.00–1.05)**	1.26 (1.02–1.56)	0.15
Residential density [1,000 residents/km^2^]	4.55	**1.07 (1.03–1.11)**	1.36 (1.13–1.64)	0.21
**MVPA minutes overall [min/day]**				
Land types [*n*]	4.12	1.00 (0.91–1.10)		
Habitat diversity [10*SHDI]	3.84	**1.15 (1.05–1.26)**	1.72 (1.22–2.41)	22.4
Slope [% rise]	1.92	**0.81 (0.67–0.99)**	0.67 (0.46–0.98)	−10.3
Intersection density [10 crossings/km^2^]	8.37	**1.06 (1.01–1.11)**	1.57 (1.05–2.36)	18.0
Residential density [1,000 residents/km^2^]	4.55	**1.13 (1.05–1.22)**	1.74 (1.23–2.47)	23.1

a *Antilogarithm values of unstandardized regression coefficients (exp^β^) and their 95% confidence intervals (CI) for univariate linear regression Model 2 adjusted for age, sex, accelerometer wear time, and perceived difficulties in walking 500 m. Values in bold; p < 0.05*.

## Discussion

Our results show that several environment variables were associated with PA on weekdays and fewer on weekend days. The positive associations of intersection and residential densities with number of PA bouts and with MVPA minutes found only for weekdays indicate that living in environments with a higher amount of infrastructure supporting outdoor mobility and with the close proximity of service destinations is especially conducive to PA on weekdays but of less relevance at weekends. However, environmental features pertaining to natural elements were more consistently related to PA irrespective of the day of the week. Higher habitat diversity in natural and green areas was associated with more MVPA time on both weekdays and weekend days. The results suggest that older people may engage in partially different activities during weekdays compared to weekends.

Previous studies support our result showing a higher likelihood of PA on weekdays in areas with higher intersection and residential densities. In those, neighborhood walkability has shown a positive, nearly statistically significant trend in the number of MVPA bouts lasting at least 10 min (15). Walking facilities, intersection density, mixed land use, and density of recreation centers were positively associated with walking for errands (19). Walking for transport, typically to destinations at least 10 min away, was positively associated with number of neighborhood amenities and also, to a certain extent, street connectivity (20). Thus, it seems that for older adults, having destinations for daily errands within walking distance may play a major role in accumulating longer lasting and brisk PA, especially on weekdays. Yet in environments providing more services, opportunities to participate in other meaningful activities such as organized activities may be greater as well.

Habitat diversity in natural and green areas was positively associated with MVPA minutes on both weekdays and weekend days as well as with PA bouts on weekdays. These results are in line with previous findings that, in older adults, proximity to or the availability of a park are positively associated with objectively measured daily MVPA (19) and self-reported leisure-time PA (9) and that several features of recreational destinations are positively associated with self-reported recreational walking (52). Among older adults with good walking capability, natural areas with higher diversity have also been related to higher self-reported PA and to perceptions of nearby nature as a motivator to go outside the home (38). However, no conclusive evidence on the associations between the availability of parks and recreation areas and self-reported active travel among older adults has previously been reported (45). Thus, based on the present results, it seems that attractive nature-based destinations close to home may be important facilitators for outdoor leisure-time and longer lasting and higher intensity exercise-type PA throughout the week among older adults.

In contrast, road network slope was negatively associated with, in particular, long-lasting PA throughout the week and with MVPA on weekend days in our study. These results are in line with earlier observations (23, 24). In addition, our earlier studies showed that perceiving hilliness as an outdoor mobility barrier predicted maladaptive walking modifications, i.e., reducing the frequency of walking or giving up walking (22) and that steeper roads in the home neighborhood predicted the development of walking difficulties (53). In the current study, the lower number of PA bouts and fewer MVPA minutes observed among older adults who live surrounded by a steep road network, supports the earlier finding that steep slopes hinder the daily walking of older adults.

In the current study, the proportion of participants with no 10-min PA bouts was higher during weekend days than weekdays. Those who were less active overall especially tended to accumulate a lower number of PA bouts and fewer MVPA minutes on weekend days than weekdays. These observations support Marshall et al. (54), who found, among those who were the most sedentary overall, that sedentary time on weekend days was greater than on an average weekday. In our study, PA ranges were narrower on weekdays than weekend days, which might suggest higher stability in PA behavior on weekdays than at weekends. Higher variability in weekend-day PA levels, as also reported by Abel et al. (4), may partly explain why fewer environmental features were related to PA levels on weekend days than weekdays.

The estimated potential effects on PA levels of high-low differences in the values of the environmental variables showed that environmental characteristics could have practical relevance for older adults' PA. Based on our estimation, the number of PA bouts was 36% greater for participants living in high vs. low residential density areas. Similarly, 26% fewer PA bouts were estimated for those living in high vs. low slope areas. Moreover, for participants living in a high habitat diversity area were calculated 72% more minutes of MVPA than for their counterparts living in a low habitat diversity area. Although considerably higher, our results parallel those of Sallis et al. (6), who, in a study on adults in 14 cities worldwide, estimated from 14 to 21% more weekly MVPA minutes for those living in neighborhoods in the highest 5% for PA-supportive environmental features (intersection and residential densities, number of parks) compared to those living in areas in the lowest 5% for these features. However, the large confidence intervals in our hypothetical estimates are a reminder that, rather than exact numbers, the associations and their directions must be considered when assessing the practical implications of these results. Furthermore, it is not possible to generalize these results to the population level, owing to the study area-specific ranges in the environment variables and a study sample consisting of older people with better than average functional capability. However, this estimation exercise shows that even within a relatively small study area, such as the one studied here, the characteristics of different neighborhood environments can vary in ways that, depending on their home location, favor some adults more than others with respect to the extent to which they support outdoor PA. However, since individual health and psychosocial factors seem to explain a larger part of older adults' PA, the contribution of environmental factors to PA levels is necessarily limited (19). Nevertheless, the environment-PA associations that we found were not notably affected by adjustments for health and socioeconomic factors. Hence, the potential effects of environmental features on older adults' PA deserve to be acknowledged, especially as even modest increases in MVPA time are beneficial, especially for the least physically active (1, 55).

The strengths of this study include the use of a population-based sample of community-dwelling people in old age. We had high quality accelerometer-measured PA data, which allowed us to use appropriate measures of outdoor PA and also investigate environment-PA associations for weekdays and weekend days separately. Detailed geospatial data on the study area enabled us to consider several environmental aspects of participants' home surroundings. A weakness is that we had no information on the actual location of PA. Hence, the accelerometer data may at least partly have accumulated from indoor activities or from PA in outdoor environments further away from home. However, moving continuously for a minimum of 10 min or being physically active at a moderate or vigorous level is more likely to take place outside than indoors. We did not take cognitive functioning into account in our analyses, although it has been suggested that cognitive capability moderates the associations between perceived environmental features and PA among older adults (56). We regarded this as unnecessary as our participants generally showed good level of cognitive capability [median 27.0 points, IQR 3.0 in the Mini-Mental State Examination (MMSE), *n* = 167]. Additionally, the rather small study sample living in one geographical area may limit the generalizability of the results to different areas.

To conclude, our results suggest that PA behavior on weekdays compared to weekend days is more closely coupled with environmental features in the vicinity of the home. Based on the differences found in the type and numbers of environmental features associated with weekday and weekend day PA, it seems reasonable to speculate that individual objectives motivating PA might underlie temporal variation in environment-PA associations. As those older adults, who are the least physically active overall, seem to engage in less PA on weekends than weekdays, it is possible that underlining the importance of establishing daily PA routines and organizing more activities that entice older people to go outside home at weekends as well as weekdays could increase their weekly PA. In addition, neighborhood environments with high walkability, attractive destinations, and routes with low gradients as enablers of higher PA, especially on weekdays, might help older people to undertake higher weekly amounts of PA. However, to develop effective interventions, more research on the temporal, spatial, and behavioral aspects of PA in older people is needed.

## Data Availability Statement

Due to ethical and legal restrictions pseudonymized datasets are available only upon request from Taina Rantanen (taina.rantanen@jyu.fi). External collaborators may use data upon agreement on the terms of data use and publication of results.

## Ethics Statement

The studies involving human participants were reviewed and approved by the ethical committee of the University of Jyväskylä, Finland. The patients/participants provided their written informed consent to participate in this study.

## Author Contributions

KK, YG, and EP conceived and designed the study. MR, TR, and EP contributed to the participant data collection. YG processed the accelerometer raw data and was responsible for describing the physical activity measures in the manuscript. KK retrieved and processed the geospatial data, conducted the statistical analyses, and was the major contributor in interpreting the results and writing the manuscript. All authors critically revised the paper and read and approved the final manuscript.

## Conflict of Interest

The authors declare that the research was conducted in the absence of any commercial or financial relationships that could be construed as a potential conflict of interest.
