# Supplementary material for: Associations of Environmental Features With Outdoor Physical Activity on Weekdays and Weekend Days: A Cross-Sectional Study Among Older People
Source: Front Public Health. 2020 Oct 30;8:578275. doi: 10.3389/fpubh.2020.578275 (PMC7661781; doi:10.3389/fpubh.2020.578275)
Supplement: Supplementary file 1 [file Data_Sheet_1.PDF]

Additional file 1: Results of linear regression analyses for Models 1, 2, 3, and 4 (n=167)

Table A, Additional file 1. Associations of environmental features with PA bouts and MVPA minutes overall for all days (n=167).

	Number of PA bouts Overall				MVPA minutes Overall			
	M1 exp$^{\beta}$ (95% CI)	M2 exp$^{\beta}$ (95% CI)	M3 exp$^{\beta}$ (95% CI)	M4 exp$^{\beta}$ (95% CI)	M1 exp$^{\beta}$ (95% CI)	M2 exp$^{\beta}$ (95% CI)	M3 exp$^{\beta}$ (95% CI)	M4 exp$^{\beta}$ (95% CI)
Land types [n]	1.01	1.01	1.01	1.02	1.02	1.00	1.02	1.03
	(0.96 - 1.07)	(0.96 - 1.06)	(0.96 - 1.07)	(0.96 - 1.08)	(0.91 - 1.14)	(0.91 - 1.10)	(0.91 - 1.15)	(0.92 - 1.15)
Habitat diversity	**1.06**	**1.05**	**1.06**	**1.06**	**1.18**	**1.15**	**1.18**	**1.19**
[10*SHDI]	**(1.01 - 1.11)**	**(1.00 - 1.10)**	**(1.01 - 1.12)**	**(1.01 - 1.12)**	**(1.06 - 1.31)**	**(1.05 - 1.26)**	**(1.06 - 1.31)**	**(1.07 - 1.32)**
Slope [% rise]	**0.86**	**0.86**	**0.86**	**0.87**	0.82	**0.81**	0.82	0.84
	**(0.77 - 0.96)**	**(0.77 - 0.95)**	**(0.77 - 0.97)**	**(0.77 - 0.97)**	(0.65 - 1.04)	**(0.67 - 0.99)**	(0.65 - 1.05)	(0.67 - 1.07)
Intersection density	1.03	**1.03**	1.02	1.02	1.05	**1.06**	1.05	1.04
[10 crossings/km^2]	(1.00 - 1.05)	**(1.00 - 1.05)**	(0.99 - 1.05)	(0.99 - 1.05)	(0.99 - 1.11)	**(1.01 - 1.11)**	(0.99 - 1.12)	(0.98 - 1.10)
Residential density	**1.07**	**1.07**	**1.07**	**1.06**	**1.12**	**1.13**	**1.13**	**1.11**
[1000 residents/km^2]	**(1.02 - 1.12)**	**(1.03 - 1.11)**	**(1.02 - 1.12)**	**(1.02 - 1.11)**	**(1.02 - 1.23)**	**(1.05 - 1.22)**	**(1.03 - 1.24)**	**(1.01 - 1.21)**

Associations adjusted for age, sex, and average accelerometer wear time on the respective days in Model 1 (M1), and additionally for perceived difficulties in walking 500 meters in Model 2 (M2), years of education in Model 3 (M3), and number of chronic conditions in Model 4 (M4). Note: Antilogarithm values of unstandardized regression coefficients (exp$^{\beta}$) and their 95% confidence intervals (CI) from univariate linear regression models show proportional effect of a one-unit increase in predictor value on the outcome variable value. Values in bold; $p < .05$

Table B, Additional file 1. Associations of environmental features with PA bouts for weekdays and weekend days (n=167).

	Number of PA bouts Weekdays				Number of PA bouts Weekend days			
	M1 exp^{β} (95% CI)	M2 exp^{β} (95% CI)	M3 exp^{β} (95% CI)	M4 exp^{β} (95% CI)	M1 exp^{β} (95% CI)	M2 exp^{β} (95% CI)	M3 exp^{β} (95% CI)	M4 exp^{β} (95% CI)
Land types [n]	1.00 (0.95 - 1.06)	1.00 (0.94 - 1.05)	1.00 (0.94 - 1.06)	1.01 (0.95 - 1.06)	1.04 (0.98 - 1.11)	1.04 (0.98 - 1.10)	1.04 (0.98 - 1.11)	1.05 (0.98 - 1.11)
Habitat diversity [10*SHDI]	**1.07 (1.02 - 1.13)**	**1.07 (1.01 - 1.12)**	**1.07 (1.02 - 1.13)**	**1.08 (1.02 - 1.14)**	1.03 (0.97 - 1.09)	1.02 (0.97 - 1.08)	1.04 (0.98 - 1.11)	1.03 (0.97 - 1.10)
Slope [% rise]	**0.86 (0.77 - 0.97)**	**0.86 (0.77 - 0.96)**	**0.87 (0.77 - 0.98)**	**0.87 (0.78 - 0.98)**	**0.86 (0.75 - 0.98)**	**0.85 (0.75 - 0.97)**	**0.86 (0.75 - 0.98)**	**0.86 (0.75 - 0.98)**
Intersection density [10 crossings/km^2]	**1.03 (1.00 - 1.06)**	**1.03 (1.01 - 1.06)**	1.03 (1.00 - 1.06)	1.03 (1.00 - 1.06)	1.01 (0.98 - 1.05)	1.01 (0.98 - 1.05)	1.01 (0.98 - 1.05)	1.01 (0.98 - 1.05)
Residential density [1000 residents/km^2]	**1.08 (1.04 - 1.13)**	**1.09 (1.04 - 1.13)**	**1.08 (1.03 - 1.13)**	**1.08 (1.03 - 1.13)**	1.03 (0.98 - 1.08)	1.03 (0.98 - 1.08)	1.03 (0.98 - 1.09)	1.03 (0.97 - 1.08)

Associations adjusted for age, sex, and average accelerometer wear time on the respective days in Model 1 (M1), and additionally for perceived difficulties in walking 500 meters in Model 2 (M2), years of education in Model 3 (M3), and number of chronic conditions in Model 4 (M4). Note: Antilogarithm values of unstandardized regression coefficients (exp^{β}) and their 95% confidence intervals (CI) from univariate linear regression models show proportional effect of a one-unit increase in predictor value on the outcome variable value. Values in bold; $p < .05$

Table C, Additional file 1. Associations of environmental features with MVPA minutes for weekdays and weekend days (n=167).

	MVPA minutes Weekdays				MVPA minutes Weekend days			
	M1 exp$^{\beta}$ (95% CI)	M2 exp$^{\beta}$ (95% CI)	M3 exp$^{\beta}$ (95% CI)	M4 exp$^{\beta}$ (95% CI)	M1 exp$^{\beta}$ (95% CI)	M2 exp$^{\beta}$ (95% CI)	M3 exp$^{\beta}$ (95% CI)	M4 exp$^{\beta}$ (95% CI)
Land types [n]	1.00	0.99	1.00	1.01	1.07	1.05	1.07	1.08
	(0.89 - 1.13)	(0.89 - 1.09)	(0.89 - 1.13)	(0.90 - 1.14)	(0.93 - 1.22)	(0.94 - 1.17)	(0.93 - 1.22)	(0.95 - 1.23)
Habitat diversity	**1.19**	**1.16**	**1.19**	**1.20**	**1.14**	**1.11**	**1.15**	**1.15**
[10*SHDI]	**(1.07 - 1.32)**	**(1.06 - 1.28)**	**(1.06 - 1.33)**	**(1.08 - 1.34)**	**(1.01 - 1.29)**	**(1.00 - 1.23)**	**(1.01 - 1.31)**	**(1.02 - 1.30)**
Slope [% rise]	0.83	0.82	0.83	0.85	0.79	**0.78**	0.79	0.81
	(0.65 - 1.06)	(0.66 - 1.01)	(0.65 - 1.06)	(0.67 - 1.08)	(0.60 - 1.04)	**(0.62 - 0.98)**	(0.60 - 1.05)	(0.62 -1.07)
Intersection density	**1.06**	**1.07**	**1.07**	1.05	1.02	1.03	1.02	1.01
[10 crossings/km^2]	**(1.00 - 1.13)**	**(1.01 - 1.12)**	**(1.00 - 1.13)**	(0.99 - 1.11)	(0.96 - 1.09)	(0.97 - 1.09)	(0.95 - 1.10)	(0.95 - 1.08)
Residential density	**1.15**	**1.15**	**1.15**	**1.13**	1.07	1.08	1.07	1.05
[1000 residents/km^2]	**(1.04 - 1.26)**	**(1.06 - 1.25)**	**(1.05 - 1.27)**	**(1.03 - 1.24)**	(0.96 - 1.19)	(0.99 - 1.18)	(0.96 - 1.20)	**(**0.95 - 1.17)

Associations adjusted for age, sex, and average accelerometer wear time on the respective days in Model 1 (M1), and additionally for perceived difficulties in walking 500 meters in Model 2 (M2), years of education in Model 3 (M3), and number of chronic conditions in Model 4 (M4). Note: Antilogarithm values of unstandardized regression coefficients (exp$^{\beta}$) and their 95% confidence intervals (CI) from univariate linear regression models show proportional effect of a one-unit increase in predictor value on the outcome variable value. Values in bold; $p < .05$
