# Supplementary material for: Associations of Environmental Features With Outdoor Physical Activity on Weekdays and Weekend Days: A Cross-Sectional Study Among Older People
Source: Front Public Health. 2020 Oct 30;8:578275. doi: 10.3389/fpubh.2020.578275 (PMC7661781; doi:10.3389/fpubh.2020.578275)
Supplement: Supplementary file 2 [file Data_Sheet_2.PDF]

Additional file 2. Results of linear regression sensitivity analyses for Models 1, 2, 3, and 4 (n=139)

Table A, Additional file 2. Associations of environmental features with PA bouts and MVPA minutes overall for all days (n=139).

	Number of PA bouts Overall				MVPA minutes Overall			
	M1 exp^{β} (95% CI)	M2 exp^{β} (95% CI)	M3 exp^{β} (95% CI)	M4 exp^{β} (95% CI)	M1 exp^{β} (95% CI)	M2 exp^{β} (95% CI)	M3 exp^{β} (95% CI)	M4 exp^{β} (95% CI)
Land types [n]	1.01 (0.95 - 1.08)	1.00 (0.95 - 1.06)	1.01 (0.95 - 1.08)	1.02 (0.96 - 1.08)	1.02 (0.90 - 1.16)	0.98 (0.89 - 1.09)	1.02 (0.90 - 1.16)	1.03 (0.91 - 1.17)
Habitat diversity [10*SHDI]	**1.07 (1.01 - 1.13)**	**1.06 (1.00 - 1.11)**	**1.07 (1.01 - 1.14)**	**1.08 (1.02 - 1.14)**	**1.21 (1.07 - 1.35)**	**1.16 (1.06 - 1.28)**	**1.21 (1.07 - 1.36)**	**1.23 (1.10 - 1.38)**
Slope [% rise]	**0.86 (0.75 - 0.98)**	**0.87 (0.77 - 0.98)**	**0.86 (0.75 - 0.98)**	**0.87 (0.76 - 1.00)**	0.76 (0.57 - 1.00)	**0.79 (0.63 - 0.98)**	0.76 (0.57 - 1.01)	0.78 (0.60 - 1.03)
Intersection density [10 crossings/km^2]	**1.03 (1.00 - 1.07)**	**1.03 (1.00 - 1.06)**	1.03 (1.00 - 1.06)	1.03 (1.00 - 1.06)	**1.07 (1.01 - 1.14)**	**1.07 (1.02 - 1.13)**	**1.08 (1.01 - 1.15)**	1.06 (1.00 - 1.13)
Residential density [1000 residents/km^2]	**1.08 (1.03 - 1.13)**	**1.07 (1.03 - 1.12)**	**1.08 (1.02 - 1.13)**	**1.07 (1.02 - 1.12)**	**1.13 (1.03 - 1.25)**	**1.13 (1.04 - 1.22)**	**1.14 (1.03 - 1.27)**	**1.12 (1.02 - 1.23)**

Associations adjusted for age, sex, and average accelerometer wear time on the respective days in Model 1 (M1), and additionally for perceived difficulties in walking 500 meters in Model 2 (M2), years of education in Model 3 (M3), and number of chronic conditions in Model 4 (M4). Note: Antilogarithm values of unstandardized regression coefficients (exp^{β}) and their 95% confidence intervals (CI) from univariate linear regression models show proportional effect of a one-unit increase in predictor value on the outcome variable value. Values in bold; $p < .05$

Table B, Additional file 2. Associations of environmental features with PA bouts for weekdays and weekend days (n=139).

	Number of PA bouts Weekdays				Number of PA bouts Weekend days			
	M1 exp^{β} (95% CI)	M2 exp^{β} (95% CI)	M3 exp^{β} (95% CI)	M4 exp^{β} (95% CI)	M1 exp^{β} (95% CI)	M2 exp^{β} (95% CI)	M3 exp^{β} (95% CI)	M4 exp^{β} (95% CI)
Land types [n]	1.00 (0.94 - 1.07)	0.99 (0.93 - 1.05)	1.00 (0.93 - 1.06)	1.01 (0.95 - 1.07)	1.04 (0.97 - 1.12)	1.03 (0.97 - 1.10)	1.04 (0.97 - 1.12)	1.05 (0.98 - 1.12)
Habitat diversity [10*SHDI]	**1.09 (1.02 - 1.15)**	**1.07 (1.02 - 1.13)**	**1.09 (1.02 - 1.16)**	**1.10 (1.04 - 1.16)**	1.03 (0.97 - 1.11)	1.02 (0.96 - 1.08)	1.05 (0.98 - 1.12)	1.04 (0.97 - 1.11)
Slope [% rise]	**0.87 (0.75 - 1.00)**	**0.88 (0.77 - 1.00)**	**0.87 (0.75 - 1.00)**	0.88 (0.77 - 1.01)	0.86 (0.73 - 1.00)	0.87 (0.75 - 1.01)	0.86 (0.73 - 1.01)	0.86 (0.74 - 1.01)
Intersection density [10 crossings/km^2]	**1.04 (1.01 - 1.07)**	**1.04 (1.01 - 1.07)**	**1.04 (1.01 - 1.07)**	**1.04 (1.00 - 1.07)**	1.01 (0.98 - 1.05)	1.01 (0.98 - 1.05)	1.01 (0.98 - 1.05)	1.01 (0.98 - 1.05)
Residential density [1000 residents/km^2]	**1.10 (1.05 - 1.15)**	**1.09 (1.05 - 1.14)**	**1.10 (1.04 - 1.15)**	**1.09 (1.04 - 1.14)**	1.02 (0.97 - 1.08)	1.02 (0.97 - 1.07)	1.03 (0.97 - 1.09)	1.02 (0.97 - 1.08)

Associations adjusted for age, sex, and average accelerometer wear time on the respective days in Model 1 (M1), and additionally for perceived difficulties in walking 500 meters in Model 2 (M2), years of education in Model 3 (M3), and number of chronic conditions in Model 4 (M4). Note: Antilogarithm values of unstandardized regression coefficients (exp^{β}) and their 95% confidence intervals (CI) from univariate linear regression models show proportional effect of a one-unit increase in predictor value on the outcome variable value. Values in bold; $p < .05$

Table C, Additional file 2. Associations of environmental features with MVPA minutes for weekdays and weekend days (n=139).

	MVPA minutes Weekdays				MVPA minutes Weekend days			
	M1 exp$^{\beta}$ (95% CI)	M2 exp$^{\beta}$ (95% CI)	M3 exp$^{\beta}$ (95% CI)	M4 exp$^{\beta}$ (95% CI)	M1 exp$^{\beta}$ (95% CI)	M2 exp$^{\beta}$ (95% CI)	M3 exp$^{\beta}$ (95% CI)	M4 exp$^{\beta}$ (95% CI)
Land types [n]	1.00	0.97	1.01	1.02	1.06	1.01	1.06	1.07
	(0.88 - 1.14)	(0.87 - 1.08)	(0.88 - 1.15)	(0.90 - 1.16)	(0.92 - 1.22)	(0.90 - 1.14)	(0.91 - 1.23)	(0.93 - 1.24)
Habitat diversity	**1.22**	**1.18**	**1.22**	**1.25**	**1.17**	**1.12**	**1.18**	**1.19**
[10*SHDI]	**(1.08 - 1.38)**	**(1.07 - 1.30)**	**(1.08 - 1.38)**	**(1.11 - 1.40)**	**(1.02 - 1.34)**	**(1.00 - 1.25)**	**(1.03 - 1.36)**	**(1.04 - 1.36)**
Slope [% rise]	0.76	0.79	0.76	0.79	0.74	0.77	0.74	0.76
	(0.57 - 1.01)	(0.62 - 1.00)	(0.57 - 1.02)	(0.60 - 1.05)	(0.54 - 1.02)	(0.60 - 1.00)	(0.53 - 1.03)	(0.56 - 1.05)
Intersection density	**1.09**	**1.08**	**1.09**	**1.08**	1.03	1.03	1.03	1.02
[10 crossings/km^2]	**(1.02 - 1.16)**	**(1.03 - 1.14)**	**(1.02 - 1.17)**	**(1.01 - 1.15)**	(0.96 - 1.11)	(0.97 - 1.09)	(0.96 - 1.12)	(0.95 - 1.10)
Residential density	**1.17**	**1.16**	**1.18**	**1.15**	1.06	1.05	1.06	1.04
[1000 residents/km^2]	**(1.05 - 1.29)**	**(1.07 - 1.26)**	**(1.06 - 1.31)**	**(1.04 - 1.27)**	(0.94 - 1.19)	(0.96 - 1.15)	(0.94 - 1.20)	(0.93 - 1.17)

Associations adjusted for age, sex, and average accelerometer wear time on the respective days in Model 1 (M1), and additionally for perceived difficulties in walking 500 meters in Model 2 (M2), years of education in Model 3 (M3), and number of chronic conditions in Model 4 (M4). Note: Antilogarithm values of unstandardized regression coefficients (exp$^{\beta}$) and their 95% confidence intervals (CI) from univariate linear regression models show proportional effect of a one-unit increase in predictor value on the outcome variable value. Values in bold; $p < .05$
